# Carcass and Meat Quality Characteristics and Changes of Lean and Fat Pigs After the Growth Turning Point

**DOI:** 10.3390/foods14152719

**Published:** 2025-08-03

**Authors:** Tianci Liao, Mailin Gan, Yan Zhu, Yuhang Lei, Yiting Yang, Qianli Zheng, Lili Niu, Ye Zhao, Lei Chen, Yuanyuan Wu, Lixin Zhou, Jia Xue, Xiaofeng Zhou, Yan Wang, Linyuan Shen, Li Zhu

**Affiliations:** 1 Farm Animal Genetic Resources Exploration and Innovation Key Laboratory of Sichuan Province, Sichuan Agricultural University, Chengdu 611130, China; 2020202019@stu.sicau.edu.cn (T.L.); ganmailin@stu.sicau.edu.cn (M.G.); zhuyan0720@stu.cwnu.cn (Y.Z.); leiyuhang@stu.sicau.edu.cn (Y.L.); yangyiting0914@stu.sicau.edu.cn (Y.Y.); 2024302141@stu.sicau.cn (Q.Z.); niulili@sicau.edu.cn (L.N.); zhye@sicau.edu.cn (Y.Z.); chenlei815918@sicau.edu.cn (L.C.); zxf93715@163.com (X.Z.); 14916@sicau.edu.cn (Y.W.); 2 State Key Laboratory of Swine and Poultry Breeding Industry, Sichuan Agricultural University, Chengdu 611130, China; 3 Key Laboratory of Livestock and Poultry Multi-omics, Ministry of Agriculture and Rural Affairs, College of Animal and Technology, Sichuan Agricultural University, Chengdu 611130, China; 4 Department of Biological Engineering, Sichuan Fisheries School, Chengdu 610041, China; 15883772124@163.com; 5 Chengdu Animal Disease Prevention and Control Center, Chengdu 610065, China; 15928035057@163.com (L.Z.); 15608031438@163.com (J.X.)

**Keywords:** pork, carcass traits, meat quality, growth turning point

## Abstract

Pork is a major global source of animal protein, and improving both its production efficiency and meat quality is a central goal in modern animal agriculture and food systems. This study investigated post-inflection-point growth patterns in two genetically distinct pig breeds—the lean-type Yorkshire pig (YP) and the fatty-type Qingyu pig (QYP)—with the aim of elucidating breed-specific characteristics that influence pork quality and yield. Comprehensive evaluations of carcass traits, meat quality attributes, nutritional composition, and gene expression profiles were conducted. After the growth inflection point, carcass traits exhibited greater variability than meat quality traits in both breeds, though with distinct patterns. YPs displayed superior muscle development, with the longissimus muscle area (LMA) increasing rapidly before plateauing at ~130 kg, whereas QYPs maintained more gradual but sustained muscle growth. In contrast, intramuscular fat (IMF)—a key determinant of meat flavor and texture—accumulated faster in YPs post inflection but plateaued earlier in QYPs. Correlation and clustering analyses revealed more synchronized regulation of meat quality traits in QYPs, while YPs showed greater trait variability. Gene expression patterns aligned with these phenotypic trends, highlighting distinct regulatory mechanisms for muscle and fat development in each breed. In addition, based on the growth curves, we calculated the peak age at which the growth rate declined in lean-type and fat-type pigs, which was approximately 200 days for YPs and around 270 days for QYPs. This suggests that these ages may represent the optimal slaughter times for the respective breeds, balancing both economic efficiency and meat quality. These findings provide valuable insights for enhancing pork quality through precision management and offer theoretical guidance for developing breed-specific feeding strategies, slaughter timing, and value-added pork production tailored to consumer preferences in the modern food market.

## 1. Introduction

Pork is the most important source of animal protein worldwide and plays a vital role in the human diet [1,2]. As living standards improve and consumer preferences change, the demand for pork is also evolving. People now not only care about quantity but also place greater emphasis on meat quality and flavor [3,4]. In today’s market, consumers increasingly prioritize the nutritional value, palatability, and safety of pork products, prompting meat quality enhancement to become a central objective in modern animal husbandry practices [5,6].

As animals grow, their carcass composition and meat quality change continuously. These dynamic changes are influenced by a complex interaction of genetics, nutrition, and physiological regulation [7,8]. In commercial production systems, the core challenge lies in achieving a balance between maximizing growth efficiency (e.g., feed conversion rate and weight gain) and ensuring desirable meat quality traits (e.g., tenderness, intramuscular fat content, and pH stability) [9,10]. This conflict becomes especially noticeable in the late fattening phase. At this stage, the growth rate naturally slows down. However, important physiological changes become more pronounced. These include increased lipid accumulation, muscle fiber maturation, and changes in water-holding capacity [11,12].

In recent years, precision feeding has emerged as a promising livestock management approach that dynamically adjusts feed supply according to the physiological status and nutritional needs of pigs [13,14]. This strategy not only enhances feed efficiency [15] and meat quality [16] but also minimizes nutrient waste and environmental impact [17]. However, the successful implementation of precision feeding requires a detailed understanding of pig growth dynamics across different developmental stages. One critical period is around the growth inflection point—the stage at which daily weight gain begins to decline. Although growth slows during this phase, significant physiological changes occur, including increased fat deposition, muscle fiber maturation, and shifts in water-holding capacity [8,18,19]. Therefore, it is important to understand how carcass and meat quality traits change around this critical time point. Such knowledge helps develop precise feeding strategies and determine the best time for slaughter. These decisions can have a direct impact on both economic benefits and the final quality of pork products. Especially in China, pork from local pig breeds with higher intramuscular fat and superior meat quality is priced 1.5 to 2 times higher than that of commercial lean-type pigs, yet consumers remain willing to purchase it.

Some studies have shown that integrating genetic selection with feeding strategies can more effectively improve pork quality and nutritional value [20]. In this study, two representative pig models were selected: Yorkshire pigs (YPs), a typical lean-type breed widely used in commercial production, and Qingyu pigs (QYPs), a traditional Chinese indigenous fatty-type breed [21,22]. Yorkshire pigs are characterized by rapid growth, high feed conversion efficiency, and lean meat yield, making them ideal for intensive farming systems. In contrast, Qingyu pigs grow more slowly and have lower lean meat percentages but are renowned for their tender texture and rich flavor, making them highly favored in local markets.

This study uses two genetically distinct pig models to investigate how carcass composition and meat quality traits change after the growth inflection point. It particularly focuses on how these developmental changes affect pork quality. Through comparative phenotypic analysis of lean-type and fatty-type pigs, we seek to uncover the breed-specific regulatory mechanisms that shape muscle growth and fat deposition—two key determinants of pork flavor, tenderness, and nutritional value. These insights are expected to not only inform genetic improvement and precision management strategies in pig farming but also contribute to the development of high-quality, differentiated pork products that better meet evolving consumer preferences and standards in the modern food industry.

## 2. Materials and Methods

### 2.1. Animals and Treatments

In this study, we utilized on-farm management data to collect body weight and average daily gain (ADG) records of Yorkshire pigs (lean-type) and Qingyu pigs (fat-type) during the fattening period starting from 50 days of age. A total of 400 female Yorkshire pigs and 126 female Qingyu pigs were included for growth data collection. Based on the calculated growth inflection points, we selected Yorkshire and Qingyu pigs that were at or beyond their respective inflection points for slaughter, in order to evaluate changes in carcass and meat quality traits after growth inflection. All pigs were divided into eight groups by body weight, with 10 kg intervals. A total of 184 female Yorkshire pigs and 73 female Qingyu pigs were slaughtered, with detailed grouping information provided in Table 1. (Due to the relatively small number of Qingyu pigs raised per batch compared to Yorkshire pigs, the number of Qingyu pigs collected and slaughtered in this study was lower than that of Yorkshire pigs. However, the sample size for both breeds was still much larger than the minimum biological replicates required for scientific research.) All animals had free access to feed and water and were raised under similar environmental conditions. The levels of crude protein, trace minerals, vitamins, and energy in the diet met or exceeded the recommendations of the National Research Council (NRC, 1998) for different production stages. The detailed nutritional composition of the feed used during the growth stage and the fattening stage for Qingyu pigs can be found in our previous study [23]. The nutritional composition of the feed for Yorkshire pigs is provided in Table S1. The pigs were fasted for 24 h prior to slaughter. After the fasting period, they were weighed to determine their body weight (BW) at slaughter. All experimental protocols and procedures are in accordance with the requirements of the Sichuan Agricultural University Ethics Committee (Chengdu, China, No. DKY-B20131403).

### 2.2. Growth Curve

The growth curves were fitted using the Logistic growth model [24], and the detailed parameters are presented in Table 2.

### 2.3. Measurement of Carcass Traits

After slaughter, the following carcass traits were measured: Carcass weight (CW): the hot carcass weight was recorded after removal of the head, viscera, feet, and tail. Dressing percentage (DP): calculated as (carcass weight/body weight at slaughter) × 100%. Body length (CL1): the linear distance from the anterior edge of the scapula to the anterior edge of the aitch bone, measured using a flexible tape. Carcass oblique length (CL2): the diagonal distance from the shoulder joint to the pubic symphysis, measured with a measuring tape. Backfat thickness was measured at four anatomical locations using a Vernier caliper: backfat1, at the thickest point of the shoulder; backfat2, between the 6th and 7th ribs; backfat3, at the thoracolumbar junction; and backfat4, at the lumbosacral junction. Loin muscle area (LMA) was determined by tracing the cross-sectional area of the longissimus dorsi muscle at the 10th–11th rib using image analysis software [25,26]. Leaf fat (LF) was collected and weighed after evisceration. Lean percentage (LP) was calculated based on carcass composition using standard predictive equations.

### 2.4. Measurement of Meat Quality

The determination of meat quality traits mainly refers to our previous article [27]. pH values were measured at 45 min (pH1) and 24 h (pH2) post mortem using a pH meter calibrated at 4 °C. Meat color parameters were determined at 45 min (L1*, a1*, b1*) and 24 h (L2*, a2*, b2*) using a Minolta CR-300 colorimeter with D65 light and a 10° observer. Drip loss (DL) was assessed according to Honikel (1998) by suspending 30–50 g *Longissimus dorsi* samples at 4 °C for 48 h [28]. Cooking loss (CL*) was calculated by comparing pre- and post-cooking weights (internal temp 70 °C). Warner-Bratzler shear force (SF) was measured at 72 h post mortem using a TA.XT Plus texture analyzer (Stable Micro Systems, Surrey, UK). Marbling was visually scored. Water-binding capacity (WBC) was evaluated as pressure-induced water loss from meat samples.

### 2.5. Chemical Composition

The crude protein (CP) was determined by the Kjeldahl method [29], and the intramuscular fat content (IMF) was determined by Soxhlet extraction [30]. Ash: residue remaining after incineration of all organic materials in a high-temperature furnace at 550~600 °C [31]. Moisture content (MC) was determined by the oven-drying method. Approximately 5 g of minced meat sample was placed in a pre-weighed aluminum dish and dried at 105 °C for 24 h in a drying oven until a constant weight was achieved. The moisture content was calculated as the percentage of weight loss relative to the fresh sample.

### 2.6. Total RNA Extraction and Real-Time Quantitative PCR

Total RNA was extracted using RNAiso reagent (Takara, Shiga, Japan), and its concentration was determined spectrophotometrically. cDNA was synthesized using the PrimeScript™ RT Reagent Kit for mRNA (Takara, Shiga, Japan), following the manufacturer’s instructions. Quantitative real-time PCR (qPCR) was conducted using TB Green^®^ Premix Ex Taq™ II (Takara, Shiga, Japan), as previously described [32]. Gene expression was calculated based on the 2-ΔΔCT method [33]. The primers used in the q-PCR process are shown in Table S2 in the Supplementary Materials.

### 2.7. Bioinformatic Analysis

Principal component analysis (PCA), clustering heatmaps, and correlation analyses were performed using the online platform SRplot (available online: https://www.bioinformatics.com.cn (accessed on 10 May 2025), a free bioinformatics visualization tool. Kyoto encyclopedia of genes and genomes (KEGG) pathway analysis was performed on the Database for Annotation, Visualization, and Integrated Discovery (DAVID) [34].

### 2.8. Statistical Analysis

All data are reported as the mean ± standard deviation (SD). Statistical analyses were performed using SPSS software (SPSS 20.0, SPSS Inc., Chicago, IL, USA). For comparisons between two groups, Student’s *t*-test was used to assess differences. For comparisons among three or more groups, one-way analysis of variance (ANOVA) was conducted, followed by Tukey’s Honestly Significant Difference (HSD) post hoc test for multiple comparisons. Differences were considered statistically significant at *p* ≤ 0.05.

## 3. Results

### 3.1. Differences in Growth and Development of Yorkshire Pigs and Qingyu Pigs

**Figure 1:**
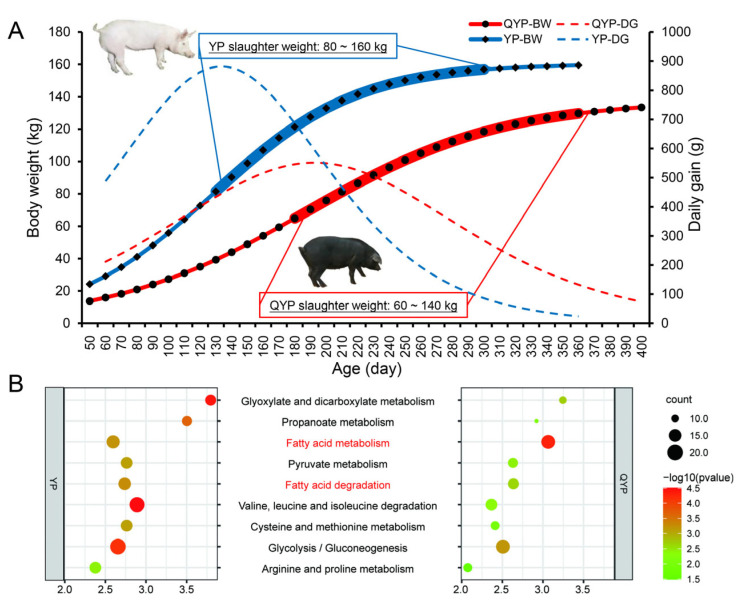
Differences in growth and development of Yorkshire pigs and Qingyu pigs. (**A**) The growth curve of YPs and QYPs. (**B**) KEGG pathway enrichment analysis of the top 500 expressed genes in the longissimus dorsi muscle of Yorkshire pigs (**left**) and Qingyu pigs (**right**).

The growth curve results revealed distinct growth patterns between the two breeds (Figure 1A and Table 2). Yorkshire pigs exhibited a faster growth rate, with a maximum body weight of approximately 160 kg, whereas Qingyu pigs grew more slowly and reached a maximum body weight of around 130 kg. Throughout the growth period, Yorkshire pigs consistently exhibited higher body weights than Qingyu pigs, indicating significantly superior growth potential. In addition, we utilized the Gene Expression Omnibus (GEO) database (NCBI, accessed on 4 Mar 2025) (accession numbers: GSE99092 [35] and GSE139322 [36]) to perform KEGG enrichment analysis based on the top 500 most highly expressed genes in the *longissimus dorsi* muscle of Yorkshire pigs and Qingyu pigs, respectively. The results revealed distinct differences in metabolic pathway enrichment between the two breeds. Yorkshire pigs showed higher enrichment in pathways related to glyoxylate and dicarboxylate metabolism, propanoate metabolism, pyruvate metabolism, glycolysis/gluconeogenesis, and fatty acid degradation, whereas Qingyu pigs were more significantly enriched in the fatty acid metabolism pathway. These findings suggest that Yorkshire pigs exhibit more active energy metabolism and lipid catabolism, which may be associated with their rapid muscle growth, while Qingyu pigs demonstrate a greater propensity for lipid deposition (Figure 1B).

The inflection point of growth in Yorkshire pigs occurred at a body weight of 80.25 kg and at 128.52 days of age, with a maximum daily gain of 882.71 g. In contrast, the inflection point for Qingyu pigs was at 68.97 kg and 187.42 days of age, with a maximum daily gain of 551.79 g. These differences in inflection points reflect the divergent growth performances between the two pig types. Based on these findings, we further selected pigs at and beyond the growth inflection points (Yorkshire: 80–160 kg; Qingyu: 60–140 kg) for slaughter to evaluate carcass traits, meat quality characteristics, and meat nutritional composition.

### 3.2. Overall Characteristics of Carcass and Meat Quality of Yorkshire Pigs and Qingyu Pigs

**Figure 2:**
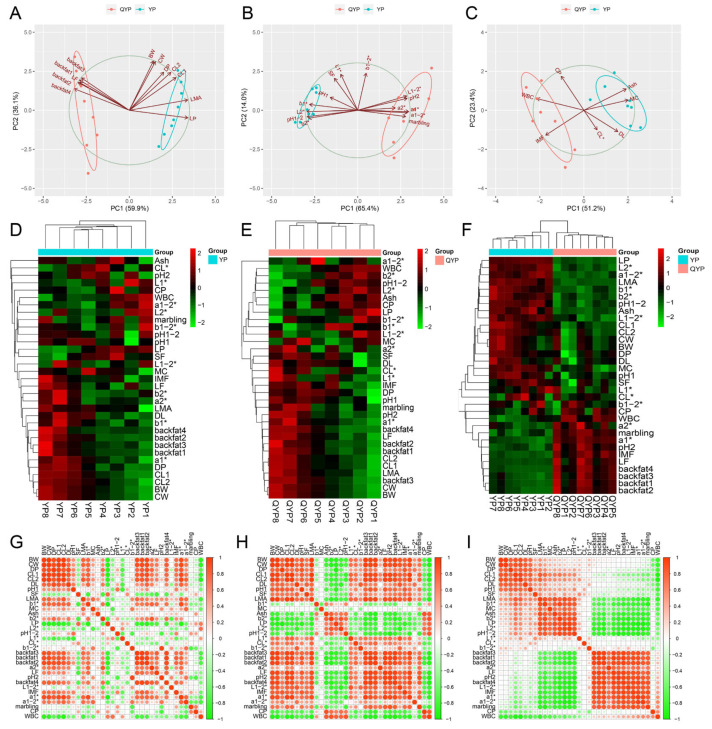
Overall characteristics of carcass and meat quality of lean and fat pigs. (**A**–**C**) Principal component analysis (PCA) of carcass traits (**A**), meat quality traits (**B**), and nutritional components (**C**) reveals distinct clustering between lean-type (Yorkshire) and fat-type (Qingyu) pigs. (**D**,**E**) Hierarchical clustering heatmaps of carcass, meat quality, and nutritional traits at different body weight stages in lean-type (**D**) and fat-type pigs (**E**). (**F**) Global clustering analysis of all traits across both breeds. (**G**,**H**) Correlation heatmaps of carcass and meat quality traits within lean-type (**G**) and fat-type pigs (**H**). (**I**) Global correlation network combining both breeds.

We slaughtered lean-type and fat-type pigs at various ages and body weights beyond their respective growth inflection points and collected data on carcass traits and meat quality parameters (Tables S3 and S4). Detailed sample numbers and grouping information are presented in Table 1. We first performed PCA to assess the overall differences between lean- and fat-type pigs in terms of carcass traits (Figure 2A), meat quality traits (Figure 2B), and nutritional composition (Figure 2C). The results revealed distinct clustering between the two breeds across all three trait categories, indicating significant differences in carcass and meat characteristics between lean- and fat-type pigs. We then conducted hierarchical clustering analyses for carcass traits, meat quality traits, and nutritional components at different body weight stages within each breed: lean-type pigs (Figure 2D) and fat-type pigs (Figure 2E). The results showed that many carcass and meat quality traits changed with increasing body weight in both breeds. However, samples from fat-type pigs exhibited clearer clustering patterns at similar body weights, whereas those from lean-type pigs were more dispersed, suggesting that trait variation in fat-type pigs followed a more gradual and consistent pattern compared to lean-type pigs. Furthermore, when clustering all measured traits across both breeds, lean-type pigs were more associated with carcass-related characteristics, while fat-type pigs were more closely associated with fat deposition traits (Figure 2F). Correlation analyses of carcass and meat quality traits within each breed revealed partially distinct correlation patterns (Figure 2G,H). In general, trait inter-correlations were weaker in lean-type pigs than in fat-type pigs, supporting the notion that phenotypic variation with increasing body weight is less pronounced in lean-type pigs. When combining data from both breeds for a global correlation analysis, we identified four major correlation clusters (Figure 2I). Strong positive correlations were observed within clusters including BW, CW, DP, CL1, CL2, DL, and pH1 and LMA, b1*, MC, Ash, b2*, LP, L2*, and pH1–2 and between backfat thickness, a2*, LF, pH2, L1–2*, IMF, a1*, a1–2*, and marbling. Notably, a strong negative correlation was found between the second and third groups—indicating that indicators such as LMA, b1*, MC, and Ash were inversely associated with fat-deposition-related traits including backfat thickness and IMF.

Overall, our findings demonstrate distinct phenotypic patterns in carcass and meat quality traits between lean- and fat-type pigs. Moreover, these traits exhibit different patterns of variation in response to increasing body weight between the two pig types.

### 3.3. The Changes in Carcass and Meat Quality Indexes of Yorkshire Pigs and Qingyu Pigs

**Figure 3:**
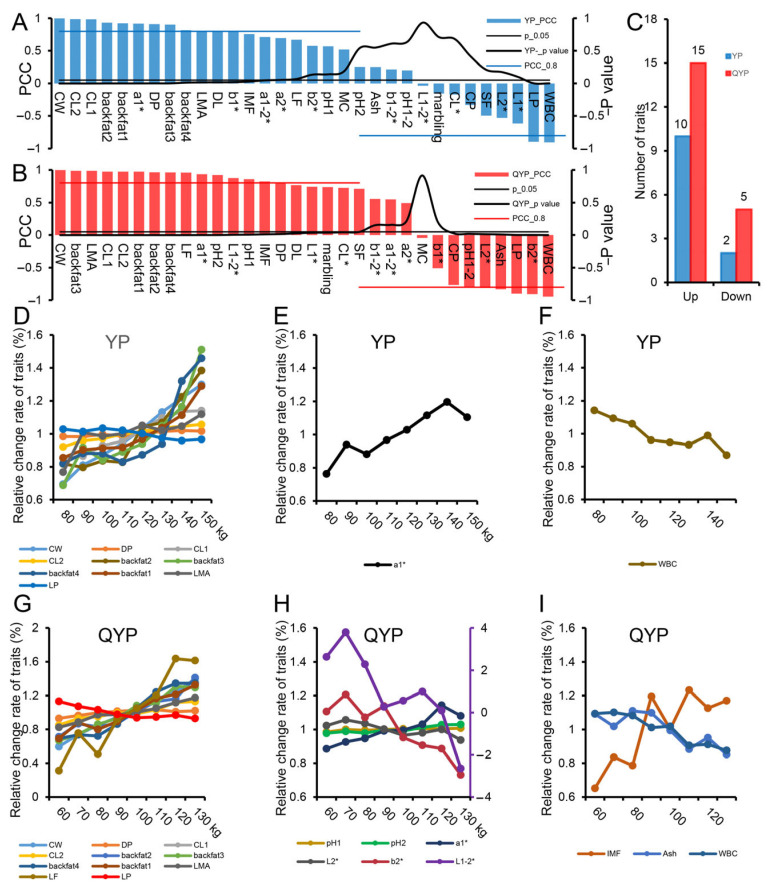
The changes in carcass and meat quality indexes of lean and fat pigs. (**A**,**B**) Identification of significantly altered carcass traits, meat quality traits, and nutritional components in lean-type (**A**) and fat-type pigs (**B**) based on significance and correlation analyses. (**C**) Summary of the number of upregulated and downregulated traits in each pig type. (**D**–**F**) Dynamic changes in carcass (**D**), meat quality traits (**E**), and nutritional composition traits (**F**) in lean-type pigs with increasing body weight. (**G**–**I**) Dynamic changes in carcass (**G**), meat quality traits (**H**), and nutritional composition traits (**I**) in fat-type pigs during growth.

We further identified significantly altered carcass traits, meat quality traits, and nutritional components in lean-type and fat-type pigs based on statistical significance and correlation analyses (Figure 3A,B). In lean-type pigs, 10 traits were significantly upregulated and 2 were downregulated; in fat-type pigs, 15 traits were significantly upregulated and 5 were downregulated (Figure 3C). Among the significantly changed traits in lean-type pigs, 10 were carcass traits. Specifically, CW, DP, CL1, CL2, backfat1, backfat2, backfat3, and backfat4 all increased with body weight, while only LP decreased as body weight increased (Figure 3D). In terms of meat quality, the a1* value increased, while water-holding capacity (WHC) decreased with growth, indicating progressive meat maturation and increased myoglobin content during development (Figure 3E,F). In fat-type pigs, 11 carcass traits were significantly altered, including CW, DP, CL1, CL2, backfat1, backfat2, backfat3, backfat4, LMA, and LF—all of which increased with body weight. Similar to lean-type pigs, LP decreased as body weight increased (Figure 3G). Six meat quality traits were significantly affected: pH1, pH2, a1*, and L1–2* increased with body weight, while L2* and b2* decreased (Figure 3H). Regarding nutritional composition, ash content and water-binding capacity (WBC) increased with growth, whereas intramuscular fat (IMF) decreased (Figure 3I).

In summary, we found that after the growth inflection point, carcass traits underwent more pronounced changes than meat quality traits in both lean- and fat-type pigs. However, the specific patterns of trait changes differed between the two pig types.

### 3.4. Differences in Muscle Development and Fat Deposition Between Yorkshire Pigs and Qingyu Pigs After Growth Inflection Point

**Figure 4:**
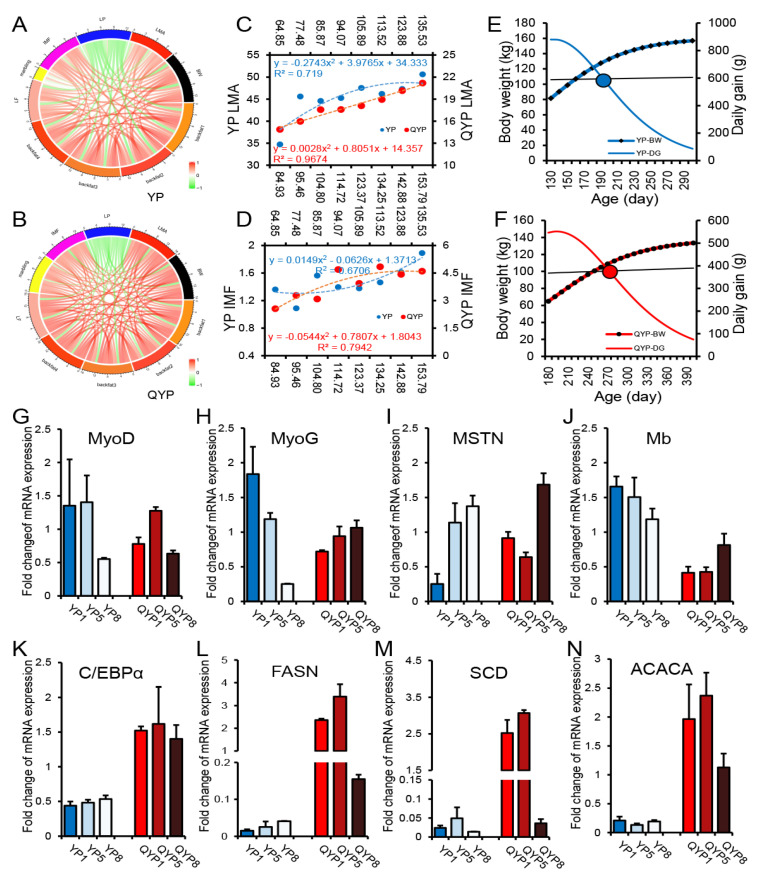
Differences in muscle development and fat deposition between lean and fat pigs after growth inflection point. (**A**,**B**) Correlation analysis of muscle growth and fat-deposition-related traits in lean-type (**A**) and fat-type (**B**) pigs. (**C**,**D**) Curve fitting analysis showing changes in LMA (**C**) and IMF (**D**) with increasing body weight in both pig types. (**E**,**F**) Peak point of growth rate decline in lean-type pigs (**E**) and fat-type pigs (**F**). (**G**–**J**) Expression patterns of muscle-development-related marker genes in muscle tissues of lean-type and fat-type pigs at different body weights. (**K**–**N**) Expression patterns of fat-deposition-related marker genes in adipose tissues of both pig types during growth.

Muscle development and fat deposition capacities are closely associated with carcass and meat quality traits in pigs. We first performed correlation analyses between traits directly related to muscle growth and fat deposition—including carcass traits, meat quality parameters, and nutritional components—in lean-type (Figure 4A) and fat-type pigs (Figure 4B). Notably, the correlation patterns differed markedly between the two types. Fat-type pigs exhibited stronger overall correlations among these traits, suggesting a more coordinated regulation of muscle and fat development compared to lean-type pigs. Subsequently, we analyzed the relationship between the LMA (longissimus muscle area) and body weight in both pig types using curve fitting (Figure 4C). In lean-type pigs, the LMA increased rapidly after the inflection point but gradually plateaued around 130 kg. In contrast, fat-type pigs showed a stable and continuous increase in the LMA beyond the inflection point. Conversely, IMF (intramuscular fat) exhibited an opposite pattern between the two pig types (Figure 4D). In lean-type pigs, the rate of IMF accumulation accelerated after the inflection point, while in fat-type pigs, IMF deposition slowed and reached a plateau around 110 kg. Although LMA growth decelerated in lean-type pigs post inflection, their absolute LMA values remained much higher than those of fat-type pigs, which would require significantly more time to reach comparable muscle area. Similarly, fat-type pigs showed much higher IMF levels than lean-type pigs. In addition, we calculated the time point at which the growth rate declined most significantly after the inflection point. The results showed that in lean-type pigs, the growth rate dropped rapidly after approximately 200 days of age, whereas in fat-type pigs, this occurred around 270 days of age (Figure 4E,F). We also quantified the expression levels of marker genes associated with muscle development and fat deposition in muscle and fat tissues. The expression profiles were consistent with observed phenotypic trends. Lean-type pigs displayed higher expression of genes related to muscle growth compared to fat-type pigs. Additionally, in lean-type pigs, the expression of the pro-myogenic genes MyoD and MyoG declined with increasing body weight, while the expression of the inhibitory gene MSTN increased; Mb (myoglobin) expression also gradually decreased. In fat-type pigs, these trends were reversed (Figure 4G–I). For adipogenic genes, fat-type pigs exhibited generally higher expression levels than lean-type pigs. However, with increasing body weight, these genes showed decreasing expression trends in fat-type pigs but increasing trends in lean-type pigs (Figure 4K–N).

In summary, lean-type and fat-type pigs demonstrated distinct capacities and regulatory patterns for muscle growth and fat deposition after their respective growth inflection points. Importantly, this does not imply that lean-type pigs lack the capacity for fat deposition or that fat-type pigs cannot improve muscle traits—rather, achieving such outcomes would require significantly extended feeding durations, which would compromise each breed’s natural advantages. Therefore, in practical production, feeding strategies and slaughter timing should be tailored to the specific growth characteristics and strengths of each pig breed.

## 4. Discussion

Previous studies have indicated that post-inflection changes in muscle growth and fat deposition are key determinants of carcass yield and meat quality in pigs [37]. Based on this, the present study systematically compared carcass traits, meat quality characteristics, and nutritional composition between a lean-type commercial breed (Yorkshire) and a fatty-type indigenous Chinese breed (Qingyu) after their respective growth inflection points. Our results showed that changes in carcass traits after the inflection point were more pronounced than those in meat quality traits for both breeds. However, the magnitude and direction of these changes displayed distinct breed-specific patterns.

Consistent with previous findings, commercial lean-type pigs typically exhibit greater muscle growth potential due to intensive selection for rapid growth [38]. In this study, YPs demonstrated a stronger capacity for muscle development, with a significantly higher LMA compared to QYPs. However, the LMA growth in YPs plateaued at approximately 130 kg body weight, whereas QYPs exhibited a steady and sustained increase in the LMA beyond the inflection point. IMF, a critical determinant of pork flavor and tenderness [39], showed opposite accumulation trends between the two breeds. In lean-type pigs, the IMF content increased rapidly post inflection, whereas in fatty-type pigs, IMF accumulation slowed and plateaued at around 110 kg. Correlation analysis revealed stronger inter-trait associations in QYPs, particularly between fat-related and meat quality traits, suggesting a more coordinated and gradual regulation of fat deposition. In contrast, YPs exhibited greater variability in muscle-related traits. Hierarchical clustering further supported these findings, showing that traits in YPs clustered more closely with carcass-related metrics, while those in QYPs were more closely associated with fat deposition traits.

At the molecular level, gene expression analysis corroborated the observed phenotypic trends. Lean-type pigs exhibited higher expression levels of muscle development–related genes (e.g., MyoD, MyoG, and Mb), which declined with increasing body weight after the inflection point. Conversely, expression of the muscle growth inhibitor MSTN increased, indicating a reduction in muscle growth potential. QYPs displayed opposite gene expression patterns, implying that they retain a capacity for continued muscle development at later stages. In terms of fat deposition, related genes were more highly expressed in QYPs, although their expression decreased as body weight increased. In YPs, however, expression of these genes gradually increased, suggesting a delayed onset of fat deposition. In addition, based on the growth curves, we calculated the time point at which the growth rate declined most significantly after the inflection point to determine the optimal slaughter age based on growth efficiency and economic benefits. The results showed that the growth rate of lean-type pigs declined significantly at around 200 days of age, while that of fat-type pigs declined at approximately 270 days of age. Therefore, these two time points were recommended as the optimal slaughter ages for the respective breeds. These findings provide a theoretical foundation for developing breed-specific feeding and slaughtering strategies to improve both production efficiency and pork quality.

## 5. Conclusions

In conclusion, our study demonstrated that lean-type (Yorkshire) and fat-type (Qingyu) pigs exhibit distinct post-inflection growth patterns in terms of carcass traits, meat quality attributes, and gene expression profiles. After the growth inflection point, carcass traits—particularly the LMA and IMF—underwent more marked changes than meat quality traits in both breeds. Yorkshire pigs showed a rapid increase in IMF content but a plateau in the LMA after reaching approximately 130 kg, while Qingyu pigs maintained continuous LMA growth and earlier IMF accumulation that plateaued around 110 kg. These breed-specific differences were also reflected at the molecular level, where lean-type pigs showed declining expression of muscle development genes and rising expression of MSTN, suggesting reduced myogenic potential. In contrast, Qingyu pigs retained active muscle development capacity and exhibited stronger correlations between fat-related gene expression and meat quality traits. Importantly, by calculating the point at which the growth rate declined most significantly after the inflection point, we identified 200 days and 270 days as the optimal slaughter ages for Yorkshire and Qingyu pigs, respectively. These time points balance growth efficiency with economic returns and meat quality outcomes. Overall, our findings suggest that tailored feeding durations and slaughter strategies based on breed-specific growth and metabolic characteristics can significantly enhance production efficiency and pork quality. These insights provide a foundation for precision pig breeding and management programs aimed at delivering high-quality, consumer-oriented pork products.

## Figures and Tables

**Table 1:** Lean and fat pig sample information.

Group		1	2	3	4	5	6	7	8
YP	BW (kg)	84.93 ± 2.80	95.46 ± 3.05	104.80 ± 2.72	114.72 ± 3.10	123.37 ± 2.83	134.25 ± 2.76	142.88 ± 3.10	153.79 ± 2.81
	ADG (g)	399.6	414.2	426.4	436	442.8	447.6	450.6	452.4
	N	8	22	37	40	20	26	17	14
QYP	BW (kg)	64.85 ± 3.70	77.48 ± 2.02	85.87 ± 2.60	94.07 ± 3.11	105.89 ± 3.33	113.52 ± 2.11	123.88 ± 3.12	135.53 ± 4.67
	ADG (g)	318.83	331.98	342.21	350.43	356.99	361.59	364.96	367.49
	N	7	6	6	17	11	6	12	8

**Table 2:** Lean and fat pig growth model parameters.

Breed	Number	PCC	A	B	K	P-BW	P-Age	MDG
YP	400	0.991	160.493	16.901	0.022	80.25	128.52	882.71
QYP	126	0.993	137.948	20.059	0.016	68.97	187.42	551.79

Note: YP: Yorkshire pig; QYP: Qingyu pig; using the growth model (Logistic growth model: $y=\frac{A}{1+Be^{-Kt}}$); Pearson: Pearson correlation coefficient; P-BW: growth inflection point for body weight, kg; P-Age: age at growth inflection point, day; MDG: maximum daily gain, g.

## Data Availability

The original contributions presented in the study are included in the article/Supplementary Material, further inquiries can be directed to the corresponding authors.

## Supplementary Materials

The following supporting information can be downloaded at https://www.mdpi.com/article/10.3390/foods14152719/s1: Table S1: The nutritional composition of the feed for Yorkshire pigs; Table S2: The primer sequences used for qRT-PCR; Table S3: Carcass, meat quality, and nutritional composition traits of Yorkshire pigs; Table S4: Carcass, meat quality, and nutritional composition traits of Qingyu Pigs.

## Abbreviations

The following abbreviations are used in this manuscript:

YP	Yorkshire pig
QYP	Qingyu pig
LMA	Longissimus muscle area
IMF	Intramuscular fat
BW	Body weight
CW	Carcass weight
DP	Dressing percentage
LF	Leaf fat
LP	Lean percentage
DL	Drip loss
CL*	Cooking loss
SF	Shear force
WBC	Water-binding capacity
CP	Crude protein
MC	Moisture content
KEGG	Kyoto encyclopedia of genes and genomes
